# Updated reference ranges for aminotransferase levels of Korean children and young adolescents based on the risk factors for metabolic syndrome

**DOI:** 10.1038/s41598-022-20104-y

**Published:** 2022-09-21

**Authors:** Young-Jun Seo, Young Suk Shim, Hae Sang Lee, Jin Soon Hwang

**Affiliations:** 1 Department of Pediatrics, Hallym University Chuncheon Sacred Heart Hospital, 77 Sakju-ro, Chuncheon, Gangwon-do 24253 Republic of Korea; 2 Department of Pediatrics, Ajou University Hospital, Ajou University School of Medicine, 164 World Cup-ro, Yeongtong-gu, Suwon, 16499 Republic of Korea

**Keywords:** Endocrinology, Health care, Medical research, Risk factors

## Abstract

We investigated the reference values of liver enzymes based on cardiometabolic risks among children and adolescents using the Korea National Health and Nutrition Examination Survey. A total of 8091 subjects aged 10–18 years were included from the data from 2007–2017. Overall, aspartate aminotransferase (AST), alanine aminotransferase (ALT), and the AST/ALT ratio varied with sex and age. AST levels tended to decrease with age, but ALT levels had a U-shaped curve, which resulted in a gradual increase in the AST/ALT ratio after age 13. The prevalence of MetS was strongly associated with elevated AST or ALT and a decreased AST/ALT ratio. The prevalence ratios of the development of MetS were also elevated in groups with high levels of AST and ALT and a low AST/ALT ratio. Particularly in the combined ALT and AST/ALT analyses, borderline-high levels also showed a high prevalence ratio of MetS. Liver enzymes were also involved in the increase in the adjusted mean values for each risk factor for MetS. Here, we provided updated reference values for liver enzymes based on the analysis between population-based data and cardiometabolic risk factors; AST, ALT and the AST/ALT ratio might be useful in the early diagnosis and treatment of MetS.

## Introduction

The rates of childhood obesity and metabolic syndrome are increasing worldwide^1^. Metabolic syndrome (MetS) is a pathophysiological state that has been used to associate overweight and obesity and their consequences such as cardiovascular diseases and diabetes mellitus. Since the National Cholesterol Education Program (NCEP) defined MetS in the adult population in 2001, subsequent studies have been conducted using modified criteria for MetS for children and adolescents and have reported an increasing prevalence of MetS. Although many different criteria resulted in difficulty estimating the exact prevalence in pediatric populations, a recent systematic review reported that the estimated prevalence of pediatric MetS ranged from 0.3–26.4% according to different geographic locations and populations^2^. In Korean studies, the prevalence of MetS has been reported to range from 5.7 to 10.9% according to the modified NCEP-ATP III criteria^3,4^. In particular, it its generally accepted that overweight and obese children have a higher risk of developing MetS than the normal population^5^. Indeed, a meta-study analysis showed that the prevalence of overweight and obese populations was high, from 24.09 to 56.32%^6^.

Hepatic involvement of MetS is commonly observed as hepatic steatosis in nonalcoholic fatty liver disease (NAFLD); pathological fat accumulation associated with chronic inflammation in the liver results in detrimental consequences, including impairment of glucose and lipid metabolism and an increase in cardiovascular events combined with oxidative stress, endothelial dysfunction and hypercoagulability^7^. Therefore, the recent current consensus recommends screening for NAFLD in children with accompanying MetS^8^. However, there is wide variability in the upper limit of normal for AST or ALT across different ages and between the sexes. The distinction of liver disease usually depends on the cutoff values that define an abnormal test, which is also critical for sensitivity and specificity^9^; this is the reason it is necessary to analyze population based data to establish reference intervals.

Several lines of studies have reported the upper limits of normal for liver enzymes in children and adolescents. In the United States, a population-based study suggested that the upper limits of normal foe ALT in metabolically normal individuals without liver disease were 26 mg/dl for boys and 22 mg/dl for girls^10^. Furthermore, in a Canadian study, the cutoff value for ALT was suggested to be 30 mg/dl in children between 1 and 12 years of age and 24 mg/dl in those between 13 and 19 years^11^. In other regions, including Europe^12,13^, the United Kingdom^14^, Mexico^15^, Sweden^16^, Iran^17^, Taiwan^18^ and China^19^, the reference ranges of liver enzymes in children were evaluated in a similar manner. However, in Korean children and adolescents, the similar studies were not population-based, and the association with MetS was not analyzed^20^ or adult data^21^.

In this study, we aimed to establish sex- and age-stratified reference intervals based on a healthy nonhospitalized pediatric population using data from KNHANES 2007–2017. We analyzed liver enzyme levels and other biochemical, physical, and social data from KNHAES to assess the association of AST, ALT and the AST/ALT ratio with MetS. Related components of MetS were also analyzed based on the normal, borderline-high and high levels of liver enzymes. Through the results of our population-based group study, we expect to add to our understanding of the roles of liver enzymes and a related ratio in MetS and provide a basis for novel liver enzyme reference ranges for children and adolescents in assessing the risk for MetS.

## Results

### Clinical characteristics of the study participants

**Figure 1:**
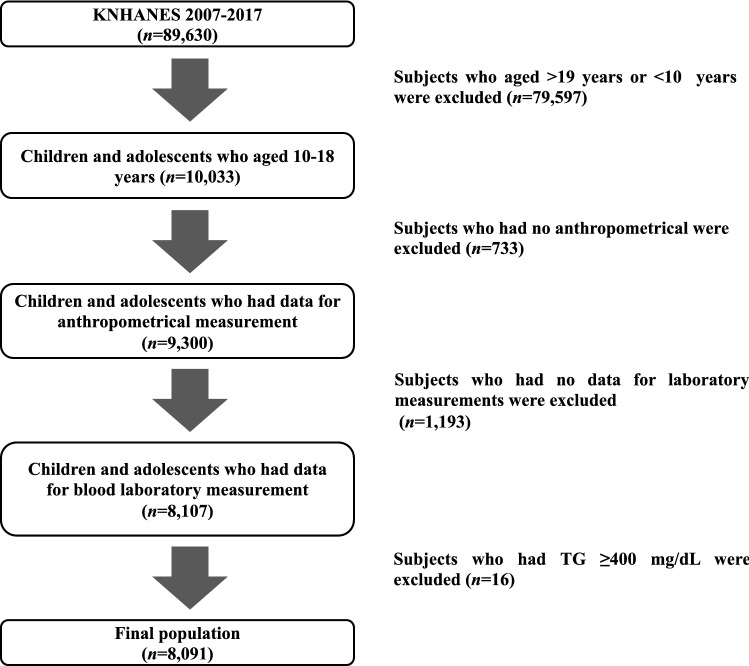
Flow chart of the study population (*n* = 8091).

Among the KNHANES from 2007 to 2017, a total of 8091 children and adolescents were selected for this study (4307 male and 3784 female) (Fig. 1). The clinical characteristics of the participants are summarized in Table 1. The mean ages of the subjects were 14.30 ± 2.51 and 14.36 ± 2.51 for males and females, respectively. There were significant sex differences in the height SDS, the WC SDS, BP, AST, ALT and the AST/ALT ratio, glucose, T-C, TG, HDL-c, LDL-c, alcohol consumption, smoking, physical activity and rural residence.

**Table 1:** Clinical characteristics of boys and girls aged 10–18 years (*n* = 8091).

	Boys	Girls	*P*
	*n* = 4307	*n* = 3784	
Age (years)	14.30 ± 2.51	14.36 ± 2.51	0.249
Height SDS	0.25 ± 1.05	0.19 ± 1.05	0.004
Weight SDS	0.10 ± 1.22	0.05 ± 1.14	0.081
Waist circumference SDS	−0.24 ± 1.13	−0.19 ± 1.09	0.028
BMI SDS	−0.04 ± 1.29	−0.05 ± 1.19	0.646
Systolic blood pressure (mmHg)	108.80 ± 10.59	104.21 ± 9.30	< 0.001
Diastolic blood pressure (mmHg)	66.38 ± 9.64	65.56 ± 8.28	< 0.001
AST (IU/L)	18.03 ± 19.96	12.16 ± 7.48	< 0.001
ALT (IU/L)	21.04 ± 10.93	17.52 ± 5.02	< 0.001
AST/ALT ratio	1.44 ± 0.61	1.61 ± 0.49	< 0.001
Glucose (mg/dL)	90.83 ± 7.98	89.43 ± 9.04	< 0.001
T-C (mg/dL)	155.80 ± 27.07	163.82 ± 26.30	< 0.001
TG (mg/dL)	49.86 ± 9.91	52.22 ± 9.86	< 0.001
HDL-c (mg/dL)	83.03 ± 47.31	86.57 ± 44.22	0.001
LDL-c (mg/dL)	89.39 ± 23.25	94.27 ± 22.89	< 0.001
Alcohol consumption (%)	1172 (27.21%)	865 (22.86%)	< 0.001
Smoking	681 (15.81%)	249 (6.58%)	< 0.001
Physical activity	2515 (58.39%)	2088 (55.18%)	< 0.001
Rural residence	700 (16.25%)	612 (16.17%)	0.004
Household income ≤ 1st quartile	467 (10.84%)	418 (11.05%)	0.947
Diagnosis of hypertension	0 (0%)	0 (0%)	> 0.999
Diagnosis of T2DM	2 (0.1%)	2 (0.1%)	> 0.999
Diagnosis of dyslipidemia	0 (0%)	0 (0%)	> 0.999

Data are presented as the mean ± standard deviation (SD).

SDS, standard deviation score; BMI, body mass index; AST, aspartate aminotransferase; ALT, alanine aminotransferase; T-C, total cholesterol; TG, triglyceride; HDL-c, high-density lipoprotein cholesterol; LDL-c, low-density lipoprotein cholesterol.

### Distribution of liver function according to age and sex

**Figure 2:**
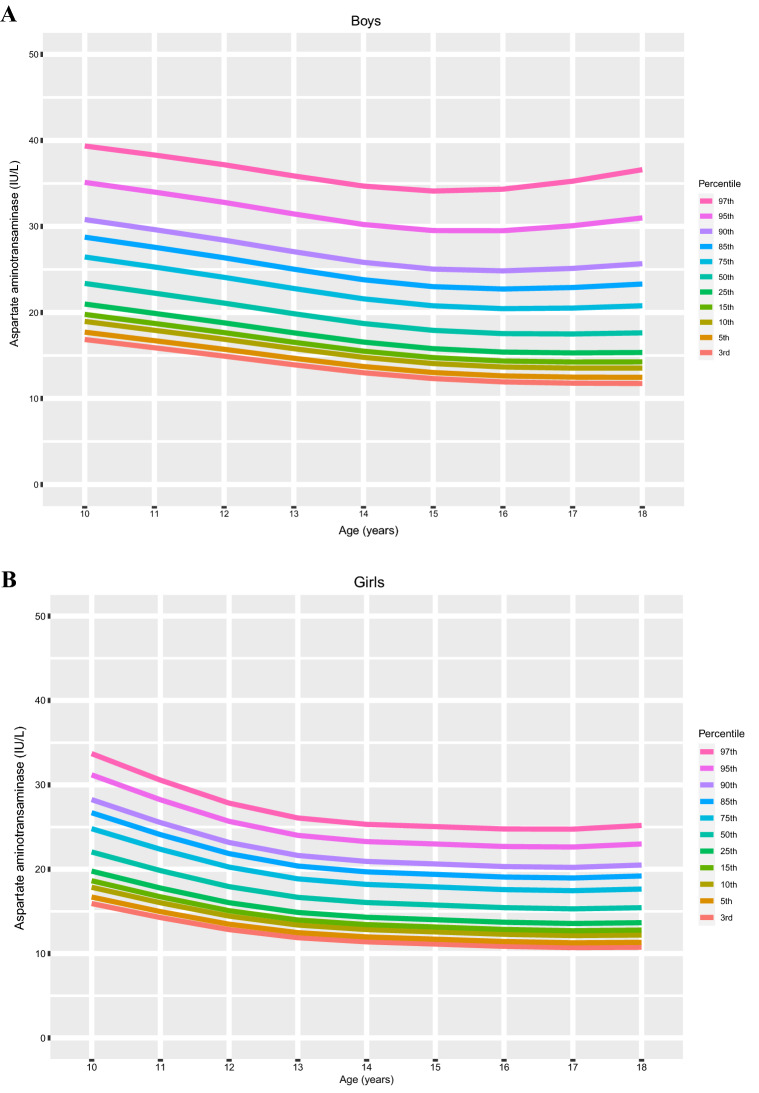

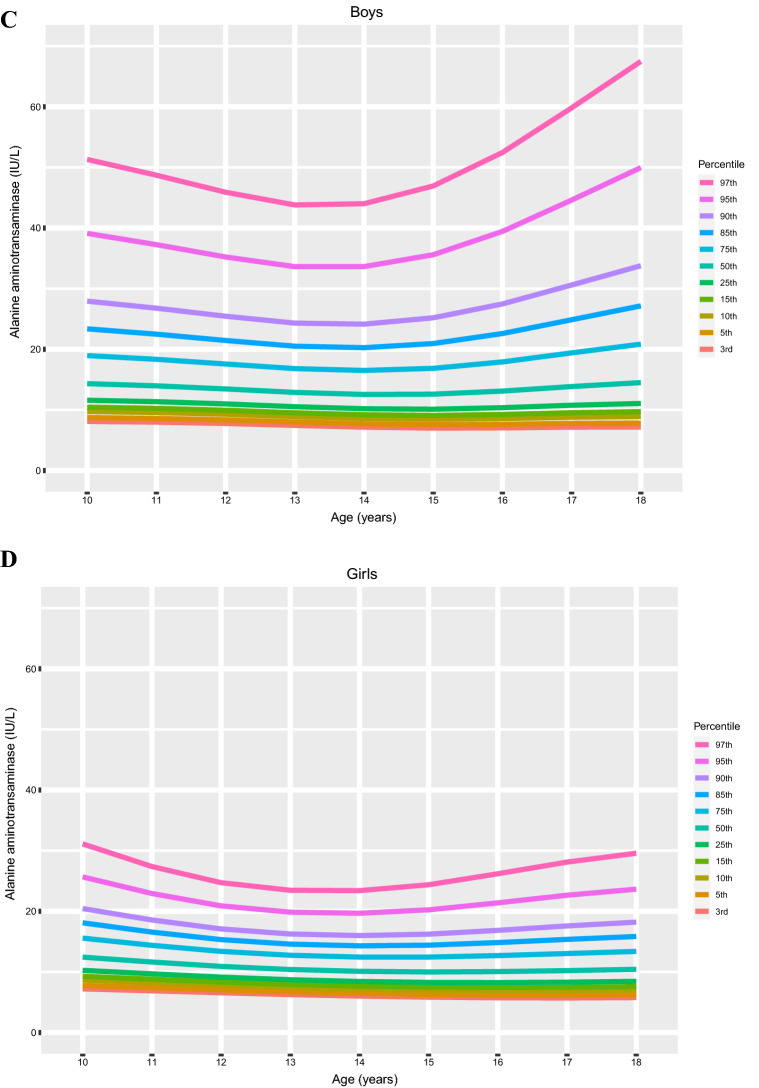

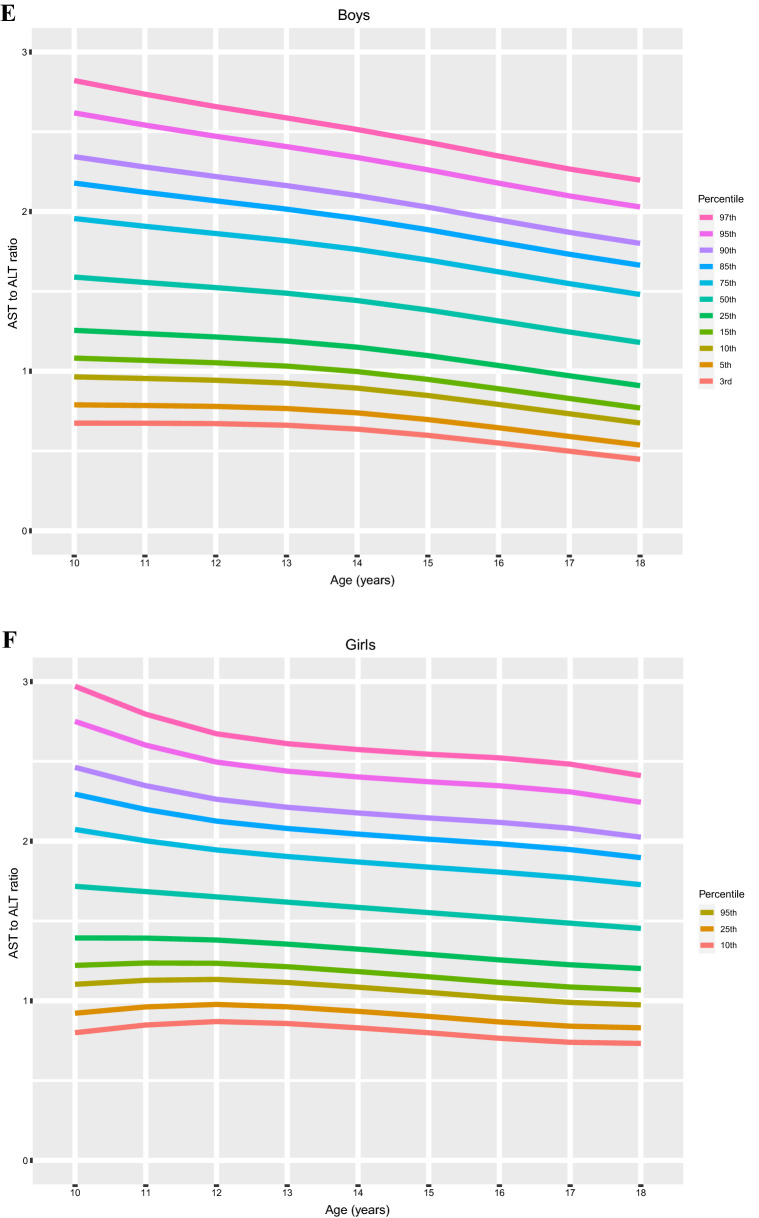
The distribution of sex- and age-specific percentiles for levels of AST, ALT, and the AST/ALT ratio in children aged 10–18 years (*n* = 8091). Each figure represents the distribution of percentile by age of (**A**) AST percentile of boys, (**B**) AST percentile of girls, (**C**) ALT percentile of boys, (**D**) ALT percentile of girls, (**E**) AST/ALT ratio percentile of boys and (**F**) AST/ALT ratio percentile of girls.

The age- and sex-specific distributions AST, ALT and the AST/ALT ratio are shown in Fig. 2. The percentile values for each age and sex, corresponding to LMS variables, are summarized in Table 2. Values of AST and ALT varied considerably according to sex and age; the percentile curve of AST tended to decrease as both boys and girls aged, but the ALT curves were slightly U-shaped in both sexes. Overall variations in AST and ALT according to age were higher in the upper percentile groups (≥ 75th percentile) than in the lower percentile groups (3rd—50th percentiles). Trends of the AST/ALT ratio decreased according to age and skewed to the left in both boys and girls; however, the levels were significantly higher in girls than in boys. Moreover, the overall AST/ALT ratio was relatively constant and decreased slightly over time.

**Table 2:** Distribution of aspartate aminotransferase (AST), alanine aminotransferase (ALT), and the AST/ALT ratio in children aged 10–18 years (*n* = 8091).

AST in boys															
Age	*n*	L	M	S	3rd	5th	10th	15th	25th	50th	75th	85th	90th	95th	97th
10	477	−1.181	23.386	0.157	16.859	17.710	18.959	19.777	20.978	23.375	26.450	28.753	30.802	35.119	39.351
11	516	−1.181	22.258	0.162	15.897	16.719	17.929	18.724	19.896	22.245	25.285	27.580	29.632	33.992	38.302
12	524	−1.181	21.102	0.168	14.928	15.718	16.887	17.656	18.793	21.088	24.082	26.359	28.409	32.797	37.173
13	543	−1.181	19.871	0.174	13.921	14.675	15.795	16.534	17.631	19.855	22.784	25.030	27.064	31.454	35.871
14	524	−1.181	18.734	0.180	12.993	13.715	14.787	15.498	16.557	18.716	21.585	23.803	25.825	30.224	34.691
15	480	−1.181	17.939	0.186	12.315	13.015	14.059	14.754	15.791	17.919	20.773	22.999	25.041	29.52	34.112
16	421	−1.181	17.562	0.192	11.931	12.625	13.663	14.356	15.394	17.539	20.441	22.726	24.835	29.501	34.329
17	427	−1.181	17.527	0.199	11.781	12.481	13.534	14.239	15.298	17.501	20.510	22.900	25.122	30.081	35.258
18	395	−1.181	17.660	0.206	11.741	12.455	13.531	14.255	15.346	17.629	20.779	23.304	25.668	30.989	36.597
All boys	4307				12.953	13.446	14.922	15.094	16.974	19.103	22.989	25.002	26.992	31.46	36.793
**AST in girls**															
10	415	−0.719	22.041	0.157	15.924	16.698	17.856	18.626	19.769	22.040	24.799	26.691	28.246	31.182	33.699
11	444	−0.719	19.836	0.160	14.268	14.970	16.022	16.723	17.763	19.836	22.361	24.097	25.526	28.232	30.559
12	431	−0.719	17.925	0.162	12.836	13.476	14.435	15.075	16.026	17.925	20.245	21.845	23.165	25.669	27.829
13	463	−0.719	16.660	0.165	11.876	12.475	13.375	13.976	14.871	16.659	18.853	20.369	21.623	24.007	26.070
14	469	−0.719	16.045	0.167	11.385	11.967	12.842	13.426	14.298	16.044	18.193	19.682	20.916	23.269	25.310
15	399	−0.719	15.747	0.170	11.122	11.698	12.564	13.143	14.009	15.747	17.891	19.382	20.620	22.986	25.046
16	414	−0.719	15.440	0.172	10.854	11.422	12.279	12.853	13.712	15.439	17.578	19.069	20.309	22.687	24.764
17	421	−0.719	15.295	0.175	10.701	11.268	12.125	12.699	13.559	15.294	17.449	18.955	20.212	22.627	24.743
18	328	−0.719	15.432	0.178	10.745	11.322	12.194	12.779	13.657	15.432	17.643	19.194	20.490	22.988	25.184
All girls	3784				11.233	12.000	13.000	13.954	14.737	16.980	19.547	21.024	22.771	25.297	27.226
**ALT in boys**															
10	477	−1.593	14.971	0.380	8.095	8.724	9.727	10.442	11.593	14.329	18.960	23.371	27.939	39.113	51.319
11	516	−1.593	14.526	0.366	7.980	8.591	9.561	10.251	11.356	13.966	18.346	22.494	26.781	37.260	48.714
12	524	−1.593	13.948	0.355	7.761	8.348	9.276	9.934	10.986	13.457	17.571	21.450	25.451	35.223	45.908
13	543	−1.593	13.349	0.353	7.442	8.003	8.892	9.521	10.526	12.886	16.810	20.507	24.319	33.629	43.809
14	524	−1.593	13.062	0.369	7.153	7.702	8.575	9.195	10.191	12.547	16.507	20.262	24.145	33.637	44.010
15	480	−1.593	13.265	0.404	6.993	7.549	8.440	9.079	10.113	12.597	16.857	20.946	25.194	35.591	46.939
16	421	−1.593	14.057	0.455	7.032	7.616	8.561	9.246	10.365	13.104	17.915	22.595	27.478	39.435	52.457
17	427	−1.593	15.290	0.525	7.149	7.771	8.790	9.535	10.769	13.854	19.405	24.875	30.599	44.608	59.821
18	395	−1.593	16.709	0.625	7.168	7.823	8.908	9.713	11.060	14.501	20.845	27.162	33.785	49.966	67.486
All boys	4307				7.818	8.000	9.000	9.848	10.994	13.045	18.192	23.317	28.001	39.472	54.059
**ALT in girls**															
10	415	−0.749	12.447	0.288	7.194	7.755	8.644	9.271	10.256	12.437	15.572	18.099	20.449	25.673	31.120
11	444	−0.749	11.638	0.274	6.886	7.403	8.218	8.789	9.680	11.631	14.385	16.566	18.566	22.931	27.384
12	431	−0.749	10.916	0.264	6.558	7.038	7.791	8.316	9.134	10.910	13.387	15.327	17.090	20.893	24.719
13	463	−0.749	10.399	0.263	6.256	6.713	7.430	7.929	8.707	10.394	12.744	14.583	16.253	19.851	23.466
14	469	−0.749	10.101	0.270	6.013	6.460	7.164	7.655	8.422	10.096	12.446	14.299	15.993	19.672	23.404
15	399	−0.749	9.997	0.282	5.833	6.282	6.990	7.488	8.269	9.990	12.446	14.410	16.227	20.235	24.377
16	414	−0.749	10.062	0.298	5.728	6.186	6.914	7.428	8.240	10.053	12.689	14.838	16.854	21.392	26.190
17	421	−0.749	10.226	0.311	5.706	6.176	6.926	7.459	8.306	10.213	13.032	15.366	17.582	22.651	28.113
18	328	−0.749	10.436	0.318	5.761	6.242	7.014	7.563	8.439	10.422	13.378	15.846	18.204	23.648	29.573
All girls	3784				6.000	6.903	7.018	8.000	8.999	10.843	13.011	15.199	17.497	22.375	26.793
**AST/ALT ratio in boys**															
10	477	0.572	1.589	0.313	0.674	0.789	0.964	1.082	1.256	1.590	1.956	2.178	2.344	2.618	2.821
11	516	0.572	1.556	0.307	0.674	0.785	0.954	1.068	1.235	1.556	1.908	2.121	2.279	2.542	2.735
12	524	0.572	1.524	0.302	0.671	0.779	0.943	1.053	1.214	1.524	1.862	2.067	2.219	2.472	2.658
13	543	0.572	1.489	0.300	0.661	0.766	0.925	1.032	1.189	1.489	1.817	2.015	2.163	2.407	2.587
14	524	0.572	1.443	0.301	0.637	0.739	0.894	0.998	1.150	1.443	1.763	1.956	2.100	2.339	2.514
15	480	0.572	1.383	0.308	0.598	0.697	0.848	0.949	1.098	1.383	1.697	1.887	2.028	2.262	2.434
16	421	0.572	1.315	0.317	0.550	0.645	0.792	0.890	1.035	1.315	1.622	1.809	1.947	2.178	2.348
17	427	0.572	1.245	0.329	0.499	0.591	0.733	0.829	0.971	1.246	1.549	1.733	1.870	2.098	2.267
18	395	0.572	1.180	0.344	0.448	0.537	0.676	0.769	0.909	1.180	1.481	1.664	1.801	2.029	2.197
All boys	4307				0.553	0.627	0.781	0.909	1.080	1.415	1.745	1.913	2.069	2.268	2.447
**AST/ALT ratio in girls**															
10	415	0.513	1.717	0.277	0.800	0.922	1.103	1.222	1.394	1.717	2.073	2.294	2.462	2.750	2.970
11	444	0.513	1.684	0.253	0.848	0.962	1.128	1.237	1.393	1.684	2.002	2.199	2.348	2.602	2.795
12	431	0.513	1.651	0.239	0.870	0.977	1.134	1.235	1.381	1.651	1.945	2.126	2.262	2.496	2.673
13	463	0.513	1.618	0.237	0.858	0.962	1.115	1.213	1.355	1.618	1.904	2.079	2.212	2.439	2.611
14	469	0.513	1.585	0.241	0.831	0.934	1.085	1.183	1.324	1.585	1.870	2.044	2.177	2.402	2.574
15	399	0.513	1.552	0.246	0.800	0.902	1.053	1.150	1.291	1.552	1.837	2.013	2.145	2.372	2.545
16	414	0.513	1.519	0.253	0.765	0.867	1.018	1.115	1.256	1.519	1.806	1.983	2.118	2.347	2.522
17	421	0.513	1.486	0.257	0.740	0.841	0.990	1.086	1.226	1.486	1.771	1.947	2.081	2.309	2.483
18	328	0.513	1.453	0.253	0.733	0.831	0.974	1.068	1.202	1.453	1.728	1.897	2.025	2.244	2.411
All girls	3784				0.767	0.885	1.047	1.152	1.303	1.576	1.876	2.001	2.159	2.392	2.603

AST, aspartate aminotransferase; ALT, alanine aminotransferase.

### Comparison of adjusted mean values of cardiometabolic risk factors according to AST, ALT, and the AST/ALT ratio

We compared the mean values of cardiometabolic risk factors, namely, WC, systolic/diastolic BP, glucose, T-C, TG, HDL-c and LDL-c stratified into normal (< 75th percentile), borderline-high (75th ≥ and < 95th percentile) and high (≥ 95th percentile) groups for AST and ALT and normal (> 25th percentile), borderline-high (> 5th and ≤ 25th percentile) and high (≤ 5th percentile) groups for the AST/ALT ratio. The upper limits of normal for boys and girls were estimated to be AST values of 23 and 20 IU/L, ALT values of 19 and 14 IU/L, and the AST/ALT ratios of 0.65 and 0.90, respectively.

The influences of AST, ALT and the AST/ALT ratio on cardiometabolic risk factors are summarized in Table 3. After adjustment for potential confounders, a comparison revealed that the adjusted mean values of T-C, TG, HDL-c and LDL-c were significantly higher in the borderline-high AST group than in the normal group. ALT levels were associated not only with lipid profiles but also with WC and BP. In the analysis of the AST/ALT ratio on cardiometabolic risk factors, the borderline-high group had a higher WC SDS, systolic BP, T-C, TG, HDL-c and LDL-c than the normal group. Moreover, the high AST/ALT ratio group had a higher WC SDS, diastolic BP, glucose, T-C, TG, HDL-c and LDL-c than the normal group, in which diastolic BP, glucose, T-C, TG and LDL-c were significantly higher than those in the borderline-high group.

**Table 3:** The adjusted means and standard errors (SE) of cardiometabolic risk factors for groups according to aspartate transaminase (AST), alanine transaminase (ALT), and the AST/ALT ratio in children aged 10–18 years (*n* = 8091).

Groups according to AST level						
	Normal	Boys			Girls	
		Borderline high	High	Normal	Borderline high	High
Waist circumference (cm)^1^	−0.31 ± 0.02	−0.22 ± 0.04	0.67 ± 0.08^b,c^	−0.15 ± 0.02	−0.37 ± 0.04^a^	−0.03 ± 0.08^c^
Systolic blood pressure (mmHg)^2^	108.74 ± 0.17	109.07 ± 0.32	108.43 ± 0.65	104.28 ± 0.17	103.88 ± 0.34	104.46 ± 0.67
Diastolic blood pressure (mmHg)^2^	66.38 ± 0.16	66.32 ± 0.30	66.72 ± 0.61	65.67 ± 0.15	65.20 ± 0.30	65.44 ± 0.60
Glucose (mg/dL)^2^	90.93 ± 0.14	90.45 ± 0.26	91.03 ± 0.53	89.45 ± 0.17	88.97 ± 0.33	91.10 ± 0.66^c^
T-C (mg/dL)^2^	153.10 ± 0.46	162.29 ± 0.86^a^	167.59 ± 1.77^b,c^	161.42 ± 0.50	171.22 ± 0.98^a^	170.06 ± 1.94^b^
TG (mg/dL)^2^	81.11 ± 0.81	85.99 ± 1.52^a^	98.58 ± 3.12^b,c^	85.02 ± 0.82	90.45 ± 1.62^a^	94.46 ± 3.21^b^
HDL-c (mg/dL)^2^	49.27 ± 0.17	51.73 ± 0.32^a^	50.51 ± 0.64	51.95 ± 0.19	52.99 ± 0.36^a^	53.18 ± 0.72
LDL-c (mg/dL)^2^	87.66 ± 0.40	93.37 ± 0.75^a^	97.36 ± 1.54^b^	92.50 ± 0.43	99.96 ± 0.86	98.14 ± 0.69
**Groups according to ALT level**						
Waist circumference (cm)^1^	−0.51 ± 0.02	0.39 ± 0.04^a^	1.26 ± 0.07^b,c^	−0.34 ± 0.02	0.17 ± 0.04^a^	0.80 ± 0.08^b,c^
Systolic blood pressure (mmHg)^2^	108.63 ± 0.17	109.40 ± 0.34	108.91 ± 0.68	104.17 ± 0.17	104.22 ± 0.34	104.71 ± 0.67
Diastolic blood pressure (mmHg)^2^	66.09 ± 0.16	67.11 ± 0.32^a^	67.94 ± 0.65^b^	65.52 ± 0.15	65.42 ± 0.31	66.80 ± 0.60
Glucose (mg/dL)^2^	90.92 ± 0.14	90.19 ± 0.27	92.17 ± 0.55^c^	89.30 ± 0.17	89.57 ± 0.34	90.97 ± 0.67^b^
T-C (mg/dL)^2^	153.13 ± 0.46	162.67 ± 0.92^a^	169.61 ± 1.87^b,c^	162.04 ± 0.49	169.05 ± 0.99^a^	171.91 ± 1.96^b^
TG (mg/dL)^2^	78.96 ± 0.80	92.93 ± 1.61^a^	106.45 ± 3.26^b,c^	83.36 ± 0.80	95.32 ± 1.63^a^	103.67 ± 3.21^b^
HDL-c (mg/dL)^2^	49.71 ± 0.17	50.44 ± 0.34	49.84 ± 0.68	52.47 ± 0.18	51.41 ± 0.37^a^	51.32 ± 0.73
LDL-c (mg/dL)^2^	87.68 ± 0.40	93.66 ± 0.80^a^	98.51 ± 1.62^b,c^	92.86 ± 0.42	98.60 ± 0.87^a^	100.08 ± 1.72^b^
**Groups according to ALT to AST ratio**						
Waist circumference (cm)^1^	−0.55 ± 0.02	0.49 ± 0.03^a^	1.11 ± 0.06^b,c^	−0.43 ± 0.02	0.27 ± 0.03^a^	1.00 ± 0.07^b,c^
Systolic blood pressure (mmHg)^2^	108.69 ± 0.17	109.19 ± 0.34	108.72 ± 0.64	104.03 ± 0.18	104.47 ± 0.31	105.39 ± 0.66
Diastolic blood pressure (mmHg)^2^	66.12 ± 0.16	66.68 ± 0.32	68.72 ± 0.60^b,c^	65.41 ± 0.16	65.62 ± 0.27	67.30 ± 0.59^b,c^
Glucose (mg/dL)^2^	90.83 ± 0.14	90.72 ± 0.27	91.29 ± 0.51	89.24 ± 0.18	89.70 ± 0.30	90.77 ± 0.65
T-C (mg/dL)^2^	153.38 ± 0.47	160.68 ± 0.93^a^	170.11 ± 1.75^b,c^	162.53 ± 0.52	166.53 ± 0.90^a^	168.82 ± 1.92^b^
TG (mg/dL)^2^	77.82 ± 0.82	96.38 ± 1.61^a^	103.50 ± 3.03^b^	83.40 ± 0.85	93.39 ± 1.47^a^	98.13 ± 3.14^b^
HDL-c (mg/dL)^2^	50.07 ± 0.17	49.28 ± 0.34	49.14 ± 0.64	52.45 ± 0.19	51.92 ± 0.33^a^	50.41 ± 0.71
LDL-c (mg/dL)^2^	87.79 ± 0.41	92.10 ± 0.81^a^	100.31 ± 1.51^b,c^	93.35 ± 0.45	95.94 ± 0.78^a^	99.07 ± 1.68^b^

The results are expressed as the mean ± standard error (SE).

WC, waist circumference; SDS, standard deviation score; BMI, body mass index; SBP, systolic blood pressure; DBP, diastolic blood pressure; T-C, total cholesterol; TG, triglyceride; HDL-c, high-density lipoprotein cholesterol; LDL-c, low-density lipoprotein cholesterol.

The normal group was classified as i) aspartate aminotransferase (AST) levels < 23 IU/L in boys, and < 20 IU/L in girls, ii) alanine aminotransferase (ALT) level < 19 IU/L in boys, and < 14 IU/L in girls, and iii) AST/ALT ratio > 1.10 in boys, and > 1.35 in girls.

The borderline-high group was classified as i) AST level ≥ 23 IU/L and 32 < IU/L in boys, and ≥ 20 IU/L and 26 < IU/L in girls, ii) ALT level ≥ 19 IU/L and < 40 IU/L in boys, and ≥ 14 IU/L and < 23 IU/L in girls, and iii) AST/ALT ratio < 0.65 and ≤ 1.10 in boys, and < 0.90 and ≤ 1.35 in girls.

The high group was classified as i) AST level ≥ 32 IU/L in boys, and ≥ 26 IU/L in girls, ii) ALT level ≥ 40 IU/L in boys, and ≥ 23 IU/L in girls, and iii) AST /ALT ratio ≤ 0.65 in boys, and ≤ 0.90 in girls.

Model 1: The adjusted means of waist circumference (WC) were estimated using analysis of covariance (ANCOVA) with Bonferroni’s *post-hoc* test after adjustment for age, alcohol consumption, smoking, physical activity, residence, household income, and diagnosis of hypertension, diabetes mellitus and dyslipidemia in the respective sex according to groups for AST, ALT and the AST/ALT ratio.

Model 2: The adjusted means of systolic blood pressure (SBP), diastolic blood pressure (DBP), glucose, total cholesterol (T-C), triglyceride (TG), high-density lipoprotein cholesterol (HDL-c), and low-density lipoprotein cholesterol (LDL-c) were estimated using analysis of covariance (ANCOVA) with Bonferroni’s *post-hoc* test after adjustment for age, body mass index (BMI) standard deviation score (SDS), alcohol consumption, smoking, physical activity, residence, household income, and diagnosis of hypertension, diabetes mellitus and dyslipidemia in the respective sexes according to groups for AST, ALT and the AST/ALT ratio.

^a^The difference was estimated between the normal group and the borderline-high group using analysis of covariance with Bonferroni’s *post-hoc* test.

^b^The difference was estimated between the normal group and the high group using analysis of covariance with Bonferroni’s *post-hoc* test.

^c^The difference was estimated between the borderline-high group and the high group using analysis of covariance with Bonferroni’s *post-hoc* test.

### Prevalence and adjusted prevalence ratios for MetS according to liver function and weight increase

**Figure 3:**
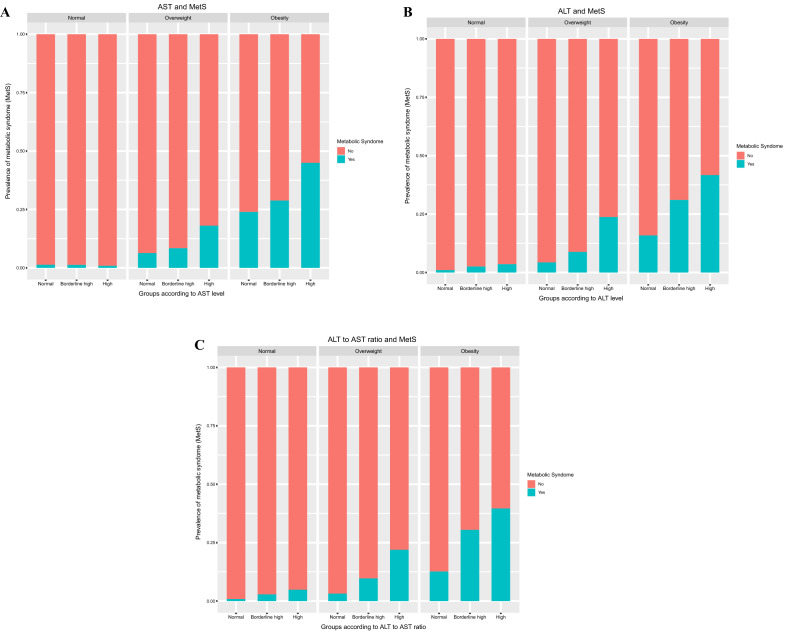
The differences in the prevalence of MetS for groups according to levels of AST, and ALT, and the AST/ALT ratio in children aged 10–18 years based on obesity (*n* = 8091). Subjects were classified as normal (< 85th percentile), overweight (≥ 85 and < 95th percentile), and obese (≥ 95th percentile) according to BMI. (**A**) The prevalence of MetS was presented according to AST, (**B**) ALT, and (**C**) the AST/ALT ratio.

**Figure 4:**
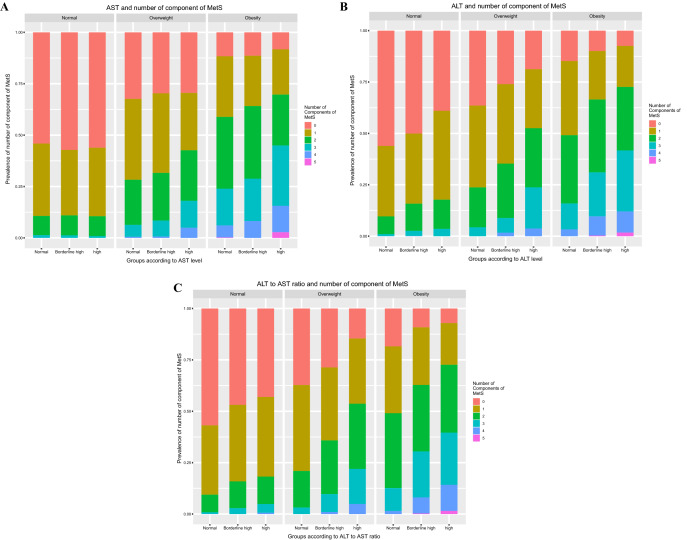
The differences in the number of MetS components for groups according to the levels of AST, and ALT, and the AST/ALT ratio in children aged 10–18 years based on the obesity (*n* = 8091). Subjects were subdivided into normal (< 85th percentile), overweight (≥ 85 and < 95th percentile), and obese (≥ 95th percentile) groups according to BMI. (**A**) The number of components of MetS was presented according to AST, (**B**) ALT, and (**C**) the AST/ALT ratio.

The prevalence of MetS was significantly increased in the overweight and obese groups as the AST level increased but not in the normal weight group. In particular, the difference was significant between the borderline-high and high groups rather than between the normal group and the borderline-high group. Although there was a sex difference regarding the prevalence ratio for some detailed components, there was no significant difference in the contribution to the overall risk increase between boys and girls. An increase in the prevalence of MetS was similarly observed in the overweight and obesity groups as ALT levels increased (Fig. 3A). The number of components of MetS, especially more than two components, also increased as AST increased in the overweight and obesity groups (Fig. 4A). In contrast to the AST analysis results, the prevalence of MetS increased even in the borderline-high group compared to the normal group as the ALT level increased (Fig. 3B). In the analysis of the number of components of MetS, the presence of more than one component increased as ALT increased in the normal, overweight and obese groups (Fig. 4B). The analysis of the AST/ALT ratio also demonstrated that the prevalence of MetS increased as the AST/ALT level decreased in the overweight and obesity groups; in contrast to the ALT values, the differences among the normal, borderline-high, and high groups were more clearly observed in the AST/ALT values (Fig. 3C). Moreover, analysis of the number of components of MetS also clearly demonstrated that more than one component increased as the AST/ALT ratio increased not only in the overweight and obesity group but also in the normal group (Fig. 4C).

The adjusted prevalence ratios for MetS and its components increased as AST and ALT increased and the AST/ALT ratio deceased, which is summarized in Table 4. Adjusted prevalence ratios for elevated glucose, elevated TG and MetS were increased for subjects with a high level of AST. Moreover, adjusted prevalence ratios for all analyzed metabolic components were considerably increased in subjects with a high level of ALT. A decreased AST/ALT ratio was most closely associated with an increased risk of MetS. In addition, the combination of high ALT and a low AST/ALT was also associated with an increased risk of elevated WC, elevated BP, elevated glucose, elevated TG, reduced HDL-c and MetS, which is summarized in Table 5.

**Table 4:** The adjusted prevalence ratio and 95% confidence intervals of MetS and its components according to the levels of aspartate aminotransferase (AST), and alanine aminotransferase (ALT), and the AST/ALT ratio in subjects aged 10–18 years (*n* = 8091).

Groups according to AST level									
	All participants			Boys			Girls		
	Normal	Borderline high	High	Normal	Borderline high	High	Normal	Borderline high	High
Elevated WC^1^	Reference	1.49 (1.24–1.80)	4.20 (3.32–5.31)	Reference	1.91 (1.48–2.55)	5.88 (4.33–7.999.84)	Reference	1.13 (0.85–1.50)	2.62 (1.80–3.81)
Elevated BP^2^	Reference	1.04 (0.94–1.16)	1.01 (0.83–1.23)	Reference	1.07 (0.91–1.26)	1.00 (0.75–1.34)	Reference	0.99 (0.86–1.14)	0.95 (0.73–1.23)
Elevated glucose^2^	Reference	1.41 (0.80–2.49)	3.65 (1.91–6.97)	Reference	1.29 (0.62–2.66)	3.05 (1.26–7.40)	Reference	1.69 (0.68–4.18)	5.20 (2.04–13.24)
Elevated TG^2^	Reference	1.19 (1.06–1.32)	1.48 (1.25–1.75)	Reference	1.59 (0.98–1.38	1.54 (1.18–2.02)	Reference	1.12 (1.01–1.26)	1.25 (1.06–1.48)
Reduced HDL-c^2^	Reference	0.88 (0.75–1.03)	0.98 (0.76–1.26)	Reference	0.74 (0.59–0.93)	0.90 (0.63–1.28)	Reference	1.10 (0.85–1.42)	1.11 (0.73–1.70)
MetS^2^	Reference	1.30 (0.98–1.74)	2.07 (1.43–2.98)	Reference	1.32 (0.92–1.90)	1.72 (1.08–2.75)	Reference	1.21 (0.74–2.00)	3.24 (1.81–5.79)
**Groups according to ALT level**									
Elevated WC^3^	Reference	4.477 (4.03–5.66)	11.03 (8.75–13.89)	Reference	6.11 (4.54–8.22)	11.15 (7.04–17.66)	Reference	3.32 (2.62–4.21)	7.53 (5.51–10.28)
Elevated BP^4^	Reference	1.18 (1.06–1.31)	1.46 (1.22–1.74)	Reference	1.28 (1.09–1.51)	1.40 (1.05–1.88)	Reference	1.02 (0.88–1.17)	1.39 (1.12–1.72)
Elevated glucose^4^	Reference	1.06 (0.58–1.94)	2.91 (1.46–5.79)	Reference	0.91 (0.40–2.08)	2.72 (1.05–7.06)	Reference	1.59 (0.67–3.78)	3.61 (1.34–9.77)
Elevated TG^4^	Reference	1.44 (1.30–1.60)	1.80 (1.55–2.10)	Reference	1.56 (1.33–1.83)	2.12 (1.65–2.71)	Reference	1.22 (1.10–1.35)	1.37 (1.18–1.59)
Reduced HDL-c^4^	Reference	1.18 (1.02–1.37)	1.38 (1.10–1.73)	Reference	0.98 (0.79–1.21)	1.11 (0.79–1.56)	Reference	1.48 (1.18–1.87)	1.75 (1.25–2.46)
MetS^4^	Reference	2.30 (1.75–3.02)	3.56 (2.54–5.00)	Reference	2.15 (1.50–3.07)	3.17 (2.02–4.97)	Reference	2.47 (1.60–3.81)	4.19 (2.48–7.10)
**Groups according to AST to ALT ratio**									
Elevated WC^3^	Reference	5.38 (4.40–6.58)	9.83 (7.12–13.58)	Reference	5.27 (3.52–7.85)	7.21 (4.14–12.54)	Reference	4.33 (3.42–5.49)	9.34 (6.57–13.29)
Elevated BP^4^	Reference	1.14 (1.03–1.26)	1.58 (1.34–1.86)	Reference	1.30 (1.09–1.51)	1.64 (1.25–2.14)	Reference	1.01 (0.89–1.15)	1.45 (1.19–1.77)
Elevated glucose^4^	Reference	0.90 (0.49–1.65)	2.20 (1.06–4.53)	Reference	0.80 (0.34–1.88)	1.90 (0.69–5.20)	Reference	1.21 (0.51–2.86)	2.80 (0.96–8.17)
Elevated TG^4^	Reference	1.46 (1.34–1.60)	1.60 (1.40–1.84)	Reference	1.76 (1.51–2.05)	2.01 (1.61–2.52)	Reference	1.20 (1.10–1.30)	1.24 (1.09–1.41)
Reduced HDL-c^4^	Reference	1.28 (1.20–1.47)	1.47 (1.20–1.82)	Reference	1.37 (1.12–1.68)	1.30 (0.94–1.80)	Reference	1.19 (0.95–1.49)	1.73 (1.26–2.38)
MetS^4^	Reference	2.90 (2.18–3.87)	4.17 (2.93–5.93)	Reference	3.30 (2.26–4.83)	4.29 (2.68–6.87)	Reference	2.40 (1.54–3.76)	4.00 (2.29–7.00)

AST, aspartate aminotransferase; WC, waist circumference; BP, blood pressure; TG, triglyceride; HDL-c, high-density-lipoprotein cholesterol; ALT, alanine aminotransferase.

The normal group was classified as i) aspartate aminotransferase (AST) level < 23 IU/L in boys, and < 20 IU/L in girls, ii) alanine aminotransferase (ALT) level < 19 IU/L in boys, and < 14 IU/L in girls, and iii) AST/ALT ratio > 1.10 in boys, and > 1.35 in girls.

The borderline-high group was classified as i) AST level ≥ 23 IU/L and 32 < IU/L in boys, and ≥ 20 IU/L and 26 < IU/L in girls, ii) ALT level ≥ 19 IU/L and < 40 IU/L in boys, and ≥ 14 IU/L and < 23 IU/L in girls, and iii) AST/ALT ratio level < 0.65 and ≤ 1.10 in boys, and < 0.90 and ≤ 1.35 in girls.

The high group was classified as i) AST level ≥ 32 IU/L in boys, and ≥ 26 IU/L in girls, ii) ALT level ≥ 40 IU/L in boys, and ≥ 23 IU/L in girls, and iii) AST/ALT ratio ≤ 0.65 in boys, and ≤ 0.90 in girls.

Model 1: The prevalence ratio and 95% confidence interval of metabolic syndrome (MetS) and its components according to groups for AST, ALT and the AST/ALT ratio were determined using multiple logistic regression after adjustment for sex, age, alcohol consumption, smoking, physical activity, rural residence, household income, and diagnosis of hypertension, type 2 diabetes mellitus (T2DM), and dyslipidemia in all participants.

Model 2: The prevalence ratio and 95% confidence interval of MetS and its components according to groups for AST, ALT, and the AST/ALT ratio were determined using multiple logistic regression after adjustment for age, body mass index (BMI) standard deviation score (SDS), alcohol consumption, smoking, physical activity, rural residence, household income, and diagnosis of hypertension, T2DM, and dyslipidemia in all participants.

Model 3: The prevalence ratio and 95% confidence interval of MetS and its components according to groups for AST, ALT and the AST/ALT ratio were determined using multiple logistic regression after adjustment for age, alcohol consumption, smoking, physical activity, rural residence, household income, and diagnosis of hypertension, T2DM, and dyslipidemia in the respective sexes.

Model 4: The prevalence ratio and 95% confidence interval of MetS and its components according to groups for AST, ALT, and the AST/ALT ratio were determined using multiple logistic regression after adjustment for age, BMI SDS, alcohol consumption, smoking, physical activity, rural residence, household income, and diagnosis of hypertension, T2DM, and dyslipidemia in the respective sexes.

**Table 5:** The adjusted prevalence ratio and 95% confidence intervals of MetS and its components according to groups according to the combination of alanine aminotransferase (ALT) and the AST/ALT ratio in subjects aged 10–18 years (*n* = 8091).

Groups according to combination of ALT and the AST/ALT ratio									
	All participants			Boys			Girls		
	Group 1^1^	Group 2^1^	Group 3^1^	Group 1^2^	Group 2^2^	Group 3^2^	Group 1^2^	Group 2^2^	Group 3^2^
Elevated WC	Reference	1.74 (1.33–2.26)	2.23 (1.57–3.18)	Reference	6.52 (4.57–9.29)	11.01 (6.48–18.69)	Reference	4.11 (3.22–5.26)	10.00 (7.34–13.63)
Elevated BP	Reference	1.12 (1.02–1.24)	1.53 (1.31–1.78)	Reference	1.22 (1.04–1.43)	1.61 (1.25–2.07)	Reference	1.00 (0.89–1.13)	1.35 (1.12–1.63)
Elevated glucose	Reference	0.99 (0.56–1.77)	2.59 (1.33–5.06)	Reference	0.75 (0.32–1.73)	2.33 (0.92–5.86)	Reference	1.52 (0.65–3.55)	3.28 (1.17–9.22)
Elevated TG	Reference	1.45 (1.32–1.59)	1.66 (1.50–1.90)	Reference	1.59 (1.37–1.86)	1.97 (1.58–2.46)	Reference	1.23 (1.12–1.36)	1.29 (1.13–1.48)
Reduced HDL-c	Reference	1.24 (1.09–1.43)	1.42 (1.16–1.74)	Reference	1.17 (0.96–1.43)	1.20 (0.88–1.63)	Reference	1.33 (1.07–1.65)	1.69 (1.24–2.30)
MetS	Reference	2.70 (2.00–3.64)	4.20 (2.95–5.96)	Reference	2.71 (1.84–4.01)	3.83 (2.40–6.12)	Reference	2.59 (1.61–4.16)	4.58 (2.62–8.01)

WC, waist circumference; BP, blood pressure; TG, triglyceride; HDL-c, high-density-lipoprotein cholesterol; ALT, alanine aminotransferase.

Group 1 was classified as the normal group based on alanine aminotransferase (ALT) level and the normal group based on the AST/ALT ratio.

Group 2 was classified as i) the borderline-high group based on ALT level and the normal group based on the AST/ALT ratio ii) i) the borderline-high group based on ALT level and the borderline-high group based on the AST/ALT ratio, and iii) normal group based on ALT levels and borderline high group based on the AST/ALT ratio.

Group 3 was classified as i) the high group based on ALT level and normal, the borderline-high, or the high group based on the AST/ALT ratio ii) the high group based on the AST/ALT ratio and the normal, the borderline-high, or the high group based on ALT level.

Model 1: The prevalence ratio and 95% confidence interval of metabolic syndrome (MetS) and its components according to groups for the combination of ALT and the AST/ALT ratio were determined using multiple logistic regression after adjustment for sex, age, body mass index (BMI) standard deviation score (SDS), alcohol consumption, smoking, physical activity, rural residence, household income, and diagnosis of hypertension, type 2 diabetes mellitus (T2DM), and dyslipidemia in all participants.

Model 2: The prevalence ratio and 95% confidence interval of MetS and its components according to groups for the combination of the AST/ALT ratio were determined using multiple logistic regression after adjustment for age, BMI SDS, alcohol consumption, smoking, physical activity, rural residence, household income, and diagnosis of hypertension, T2DM, and dyslipidemia in the respective sexes.

## Discussion

We found that clear differences in AST, ALT, and AST/ALT ratio curves between age- and sex- different subgroups; the AST levels tended to decrease with age, but the ALT curves were U-shaped, which resulted in trends of the AST/ALT ratio to reduce with age and skewed to the left. In the cardiometabolic risk assessment based on our novel references, components of MetS significantly increased in high levels of AST, ALT, and AST/ALT ratio; some of the components were increased even in borderline high levels of liver enzymes in the analysis of ANCOVA with correction of age, sex, BMI, etc. Similarly, the odds ratio of metabolic risk factors also increased in the high AST, ALT, and the low AST/ALT ratio; some components of MetS were significantly increased even in borderline high levels.

In our findings, AST and ALT were higher in boys than in girls and differed over time; AST tended to decrease with age but ALT levels were U-shaped and increased with age. These tendencies were consistent with previous reports in Korean subjects^20^ and those of other ethnicities; for instance, an obvious increase in ALT levels was observed in males after the age of 11 years^19^, and a sex difference with a continuous decrease in concentration from ages 2 to 14 years was observed for AST^11,19,22,23^. Percentile distributions of the AST/ALT ratio showed sex differences across all observed ages in our data; the ratio was significantly higher in girls than in boys. Despite conflicts with the data of the HELENA study^12^, our results were similar to those of a recent Chinese study^24^, which seems to be due to ethnic or geographic differences.

Regarding MetS, an increase in the prevalence of MetS according to AST, ALT, and the AST/ALT ratio was more clearly observed as BMI increased (Figs. 3 and 4). This result suggested that although a strong correlation exists between high levels of AST, ALT, and the AST/ALT ratio and most cardiometabolic risk factors exist, the interpretation of liver markers in children and adolescents could be adapted differentially depending on the differential BMI values. Previously, AST or AST/ALT ratios were known to indicate progression to diabetes^25^. Similarly, in our findings, high serum AST, ALT, and the AST/ALT ratio were all associated with a high prevalence ratio of elevated glucose (Table 4). These results strongly supported that a decreased AST/ALT ratio might effectively predict increased cardiometabolic risk, especially insulin resistance when high ALT levels exist. Of interest, our results showed that the associations of AST, ALT, and the AST/ALT ratio with cardiometabolic risk factors were significant both in boys and girls, which partially conflicts with a previous European study^12^. The differential distribution of overweight or obese children in the study population might affect the analysis. In addition, differential body adiposity might also influence screening tests of liver markers. Therefore, detailed comparative studies on this point will be needed in future studies.

Since the importance of childhood MetS on adulthood transition has been raised, several studies have suggested various criteria for the evaluation of MetS; however, there are still controversies regarding the establishment of uniform diagnostic criteria^2^. Among them, the modified NCEP-ATP III criteria^26–28^ adapted in our study and the International Diabetes Federation (IDF) criteria^29^ are the two most widely used definitions. The characteristic differences in the IDF criteria on MetS were the presence of obesity as a mandatory condition for the diagnosis and the cutoff value > 150 mg/dL of high TG compared to our diagnostic definition^30^. If we followed the IDF criteria, the prevalence of MetS according to liver enzyme levels in normal or overweight subjects would have been excluded in the metabolic syndromes. However, the prevalence of metabolic syndrome and its risk factors according to liver enzymes were also significantly higher even in the overweight group as in the obesity group in our results (Figs. 3 and 4). Although it is not yet clear what the exact contribution of metabolic risk factors is to the development of CVD and T2DM in adulthood, these findings might support that nonobese and overweight pediatric subjects with metabolic risk factors are also potentially at risk for adult metabolic syndromes. Therefore, detailed long-term observational and cohort studies on this point will be needed in future studies.

Based on the percentile distribution, we suggested that the upper limits of normal of AST, ALT and the AST/ALT ratio were 23 IU/L and 20 IU/L (< 75th percentile), 19 IU/L and 14 IU/L (< 75th percentile), and 1.1 and 1.35 (< 25 percentile) in boys and girls, respectively. Similar studies with non-overweight adolescents proposed sex-specific thresholds for ALT levels < 25 IU/L in males and < 22 IU/L in females to detect pediatric chronic liver disease^10^. However, Labayen et al. suggested upper limits of normal for ALT of 24–25 (75th percentile) and 22–24 IU/L (75th percentile) and thresholds of the AST/ALT ratio associated with high cardiometabolic risk of 1.0–0.74 and 0.86–0.87 (ranging from 13-35th percentile) for boys and girls, respectively^12^. In those studies, the estimated upper limits of normal of ALT in adolescents were higher than those in our report. This might be due to differences in ethnicities between the studies; other possibilities are differences in the proportion of subjects with obesity and severity of central adiposity levels. Although direct comparison of the AST/ALT ratio between our data and previous reports is difficult, it seems that stricter levels of the AST and ALT should be applied for Korean adolescents, especially for the overweight or obese subgroup for the precise estimation of cardiometabolic prognosis.

The main limitation of this study is the cross-sectional nature of the analysis, which cannot identify the temporal association of MetS with AST, ALT, and the AST/ALT ratio. A large population-based, longitudinal cohort study could address this limitation in the future by serial measurements of liver enzymes and follow-up for the occurrence of cardiometabolic events. The other limitation is that our data were from subjects of one ethnicity in a single country. Thus, comparisons and meta-analyses with other ethnic groups will be needed for the broad application of pediatric reference intervals. Despite information about a family history of premature coronary heart disease, we could not exclude familial hyperlipidemia during subject selection due to limitations on laboratory tests. Combined familial hyperlipidemia frequently accompanies NAFLD in approximately 49–76% of cases^31^, which implies that the possible effects of these comorbidities were not completely excluded in our data. Other liver markers such as gamma-GT have also been suggested to be strong predictors of cardiovascular disease and T2DM in adults^32^, and metabolic risks in adolescents^12^; however, we did not analyze other possible markers in our current study. The definition of physical activity used in this study was not consistent with the current recommendations of The American Academy of Pediatrics (AAP); a daily moderate to vigorous intensity of exercise for more than 60 min^33^. This is due to the difference in the KHANES protocol and needs improvement in future investigations.

In conclusion, we newly established reference values for AST, ALT, and the AST/ALT ratio based on the risk assessment of MetS components. High levels of AST and ALT and a low AST/ALT ratio were closely associated with the prevalence of MetS and its components. In particular, overweight and obese children and adolescents have a considerably higher prevalence of MetS and its components when liver enzymes exceed the upper limits of normal than do normal subjects. Both ALT and the AST/ALT ratio were effective in screening for metabolic risk in both sexes in a Korean population. Therefore, the age- and sex-specific reference values provided in this study may contribute to the early diagnosis and treatment of MetS.

## Materials and methods

This study was based on data from the KNHANES. The KNHANES is a cross-sectional, nationally representative survey that is conducted by the Korean Centers for Disease Control and Prevention (KCDC) annually. The survey consists of a health questionnaire, health examination, and nutritional assessment. Every participant in the KNHANES gave informed consent at the time of data collection and the survey protocols were approved by the KCDC Institutional Review Board. KNHANES was performed in accordance with relevant guidelines and regulations of the National Health Promotion Act in Korea. The database is available to the public at the KNHANES website (http://knhanes.cdc.go.kr) and details of the KNHANES have been described previously^34^. This study was also approved by the Institutional Review Board of Hallym University Chuncheon Sacred Heart Hospital (IRB No. CHUNCHEON 2021–10-004). All methods were performed in accordance with the relevant guidelines and regulations.

### Subjects

The subjects in this study were from the KNHANES from 2007 to 2017. The initial subjects comprised 10,033 children and adolescents among a total of 89,630 subjects. Subjects who had missing anthropometric data (n = 733) and those missing blood laboratory results or had any type of hepatitis were excluded (n = 1193). Subjects with abnormal triglyceride (TG) levels (≥ 400 mg/dL) (n = 16) were excluded because low-density lipoprotein cholesterol (LDL-c) levels were calculated with Friedewald’s Equation^35^.

### Measurements

Anthropometric data and blood pressure (BP) were measured by trained experts according to standardized protocols. Details of the anthropometric measurements have been described previously^26^. Standard deviation scores (SDSs) were used for height, weight, BMI, and WC which were calculated with the LMS methods using the 2017 Korean reference^36^. Blood samples were collected after the participants fasted for at least 8 h. Samples were immediately centrifuged, transported to a central laboratory (NeoDin Medical Institute, Seoul, Korea) and analyzed within 24 h. Serum levels of total cholesterol (T-C), high-density lipoprotein cholesterol (HDL-c), TG, and glucose were measured enzymatically using a Hitachi 7600 automatic analyzer (Hitachi, Tokyo, Japan). LDL-c was calculated with Friedewald’s equation^35^.

### Collection of lifestyle parameters and socioeconomic status

Lifestyle-related parameters and socioeconomic information were collected by questionnaires. Smoking was defined as having smoked more than five packs of cigarettes throughout one’s lifetime. Alcohol consumption was defined as at least two alcoholic beverages/month during the previous year. Physical activity was defined as: (1) intense physical activity for 30 min at least three days/week, (2) moderate physical activity for 30 min at least five days/week, or (3) walking for 30 min at least five days/week. Based on physical activity, subjects were classified as exercising or non-exercising. For socioeconomic information, household income was categorized as being within the lowest quartile or not. The residence area was divided into urban and rural areas.

### Definitions of MetS and its components

Obesity and overweight were defined as ≥ 95th and ≥ 85th but < 95th percentile, respectively, as shown in a previous study^36^. The definition of MetS followed the modified criteria of the National Cholesterol Education Program Adult Treatment Panel III (NCEP-ATP III), as previously described in our published paper^26^; subjects who met 3 of the following 5 criteria were defined as having MetS: (1) increased WC based on the Korean pediatric population; ≥ 90th percentile for age and sex according to the 2017 Korean growth chart^36^; (2) elevated BP, namely, SBP or DBP ≥ 90th percentile for age- and sex-matched reference data from the Korean pediatric population^36^ or treatment with antihypertensive medication; (3) fasting blood glucose ≥ 100 mg/dL or treatment for type 2 diabetes mellitus (T2DM); (4) elevated TG (≥ 110 mg/dL); and (5) low HDL-c (< 40 mg/dL). T2DM was diagnosed satisfying one or more of the following criteria: (1) subjects who self-reported their disease using a questionnaire, (2) current medication or insulin use to manage T2DM, or (3) subjects with a fasting glucose level ≥ 126 mg/dL during the survey period.

### Statistical analysis

The basic characteristics consisted of continuous variables and categorical variables; each variable is presented as the mean ± standard deviation (SD) and frequencies or percentages (%), respectively. Student’s t-test was used to compare the means of the demographic and biochemical characteristics. The chi-square (χ^2^) test was used to compare clinical categorical variables between boys and girls.

We obtained percentile curves of AST, ALT and the AST/ALT ratio as a function of age as a continuous variable, stratified by sex using the LMS model to fit smoothed L (skew), M (median), and S (coefficient of variation) curves using the General Additive Model for Location Scale and Shape (GAMLSS) package version 4.2.6. of the R statistical package. The Box–Cox Cole and Green, gamma or inverse Gaussian distributions were fitted to the observed distribution of AST, ALT and the AST/ALT ratio. Percentile curves were generated for the 3rd, 5th, 10th, 15th, 25th, 50th, 75th, 85th, 90th, 95th and 97th percentiles. The adjusted mean values of cardiometabolic risk factors were compared between three groups (normal < 75th; borderline high, ≥ 75th and < 95th; and high, ≥ 95th percentile) using analysis of covariance (ANCOVA) followed by Bonferroni’s post-hoc test after adjusting for sex, age, BMI SDS, alcohol consumption, smoking, physical activity, residence, household income, and diagnosis of hypertension, T2DM, and dyslipidemia. We estimated the adjusted prevalence ratios and 95% confidence intervals (CIs) for MetS among the normal, borderline high and high groups by multiple logistic regression analysis. The estimates were adjusted for sex, age, BMI SDS, alcohol consumption, smoking, physical activity, residence, household income, diagnosis of hypertension, T2DM, and dyslipidemia. P < 0.05 was considered indicative of statistical significance. All statistical analyses in this study were performed using R statistical package version 3.5.1 (The R Foundation for Statistical Computing, Vienna, Austria). The prevalence ratios were calculation using the prLogistic package. The prLogistic package was implemented under the FLOSS (Free/Libre Open Source Software) paradigm in the R system for statistical computing (R Development Core Team 2021) and it was available from the Comprehensive R Archive Network (CRAN) at https://cran.r-project.org/package=prLogistic and the experimental updates at GitHub repository https://github.com/Raydonal/prLogistic.

## Data Availability

The data that support the findings of this study are available in [the KNHANES website] at [https://knhanes.kdca.go.kr]. The data that support the findings of this study are available from the corresponding author, [Shim YS], upon reasonable request.

## Abbreviations

ALT: Alanine aminotransferase
AST: Aspartate aminotransferase
BP: Blood pressure
BMI: Body mass index
CI: Confidence interval
HDL-c: High-density lipoprotein cholesterol
KCDC: Korean Centers for Disease Control and Prevention
KNHANES: The Korea National Health and Nutrition Examination Survey
LDL-c: Low-density lipoprotein cholesterol
MetS: Metabolic syndrome
NAFLD: Nonalcoholic fatty liver disease
NCEP-ATP III: National Cholesterol Education Program Adult Treatment Panel III
NCEP: National Cholesterol Education Program
SDS: Standard deviation scores
T2DM: Type 2 diabetes mellitus
TG: Triglyceride
T-C: Total cholesterol
WC: Waist circumference
